# Deficiency of the TLR4 analogue RP105 aggravates vein graft disease by inducing a pro-inflammatory response

**DOI:** 10.1038/srep24248

**Published:** 2016-04-07

**Authors:** Anouk Wezel, Margreet R. de Vries, Johanna M. Maassen, Peter Kip, Erna A. Peters, Jacco C. Karper, Johan Kuiper, Ilze Bot, Paul H. A. Quax

**Affiliations:** 1 Division of Biopharmaceutics, LACDR, Leiden University, Leiden, The Netherlands; 2 Department of Surgery, Leiden University Medical Center, Leiden, The Netherlands; 3 Einthoven Laboratory for Experimental Vascular Medicine, Leiden, The Netherlands.

## Abstract

Venous grafts are often used to bypass occlusive atherosclerotic lesions; however, poor patency leads to vein graft disease. Deficiency of TLR4, an inflammatory regulator, reduces vein graft disease. Here, we investigate the effects of the accessory molecule and TLR4 analogue RadioProtective 105 (RP105) on vein graft disease. RP105 deficiency resulted in a 90% increase in vein graft lesion area compared to controls. In a hypercholesterolemic setting (LDLr^−/−^/RP105^−/−^ versus LDLr^−/−^ mice), which is of importance as vein graft disease is usually characterized by excessive atherosclerosis, total lesion area was not affected. However we did observe an increased number of unstable lesions and intraplaque hemorrhage upon RP105 deficiency. In both setups, lesional macrophage content, and lesional CCL2 was increased. *In vitro*, RP105^−/−^ smooth muscle cells and mast cells secreted higher levels of CCL2. In conclusion, aggravated vein graft disease caused by RP105 deficiency results from an increased local inflammatory response.

Rupture of an atherosclerotic lesion with subsequent thrombus formation may lead to distal embolization of the blood vessel, resulting in adverse cardiovascular events^1^. Restoring blood flow to the ischemic tissue is therefore crucial, which can be accomplished by interventions such as placement of a (drug eluting) stent or a venous graft. Vein grafts are often used because of their long length, making it possible to bypass multiple atherosclerotic lesions, and their easy accessebility^2^,^3^,^4^. However, after ten years only an approximate 40% of the grafts is still patent^5^,^6^,^7^,^8^. It is thus of high importance to elucidate the mechanisms of vein graft disease and identify new therapeutic targets to prevent vein graft failure.

Excessive and uncontrolled smooth muscle cell (SMC) accumulation and proliferation result in the formation of intimal hyperplasia^9^. This process is accompanied by leukocyte influx into the vessel wall, which aggravates the inflammatory process and leads to superimposed atherosclerosis. Late vein graft failure may eventually be the result of complete occlusion caused by intimal hyperplasia and accelerated atherosclerosis or rupture of the vein graft lesion^10^.

To study the complex mechanisms behind vein graft disease, as well as to explore possible treatment options, a previously described murine vein graft model has been used^11^. In this model, lesions display typical concentric hyperplasia as well as lesional disruptions with intraplaque hemorrhage, with high resemblance to the complex lesions present in human vein grafts. When vein grafts are placed in mice on a high cholesterol diet, superimposed atherosclerosis will add to the lesional burden, as illustrated by lipid depositions and foam cell accumulation^12^. Taken together, excessive SMC proliferation, lipid accumulation and an enhanced inflammatory response seem to be causing vein graft disease. Highlighting the importance of vascular inflammation in vein graft disease, we have previously shown that local silencing of Toll like receptor 4 (TLR4) significantly reduces vessel wall thickening in these murine venous grafts^13^, rendering this pathway of interest for future therapeutic interventions.

TLR4 belongs to the TLR family, a type of pattern recognition receptors, capable of inducing potent inflammatory signalling. A unique feature of TLR4, compared to other TLRs, is that is does not directly bind to its ligands, but is dependent on the ligand binding adaptor molecule MD2 and co-receptor CD14^14^. An additional accessory molecule known to regulate TLR4 signalling is RadioProtective 105 (RP105). Similar to TLR4, the TLR4 analogue RP105 forms a complex with the adaptor molecule MD1, but in contrast, the RP105/MD1 complex does not have an intracellular Toll/interleukin 1 receptor (TIR) signalling domain^15^. Originally, RP105 was described as a B cell specific surface molecule, capable of enhancing cellular proliferation and activation^15^. However, RP105 was later on demonstrated to be expressed on the cell membrane of dendritic cells (DCs) and macrophages as well. In contrast to its role on the B cell, RP105 inhibits TLR4 mediated responses in DCs and macrophages, leading for instance to an aggravated inflammatory response after lipopolysaccharide (LPS) injection in RP105 deficient mice^16^.

Taking into account the profound regulatory effects of RP105 on TLR4, and the previous finding that TLR4 signalling aggravates vein graft disease, it is compelling to investigate the role of RP105 in vein graft disease. Therefore, in the current study we used the murine vein graft model to establish whether RP105 deficiency affects lesion formation. We hypothesized that lack of RP105, via increased TLR4 signalling, will result in aggravated vein graft disease. Also, we aimed to determine how RP105 affects vein graft disease in a hypercholesterolemic setting with superimposed atherosclerosis.

## Results

### RP105^−/−^ mice show aggravated vein graft lesion development

**Figure 1:**
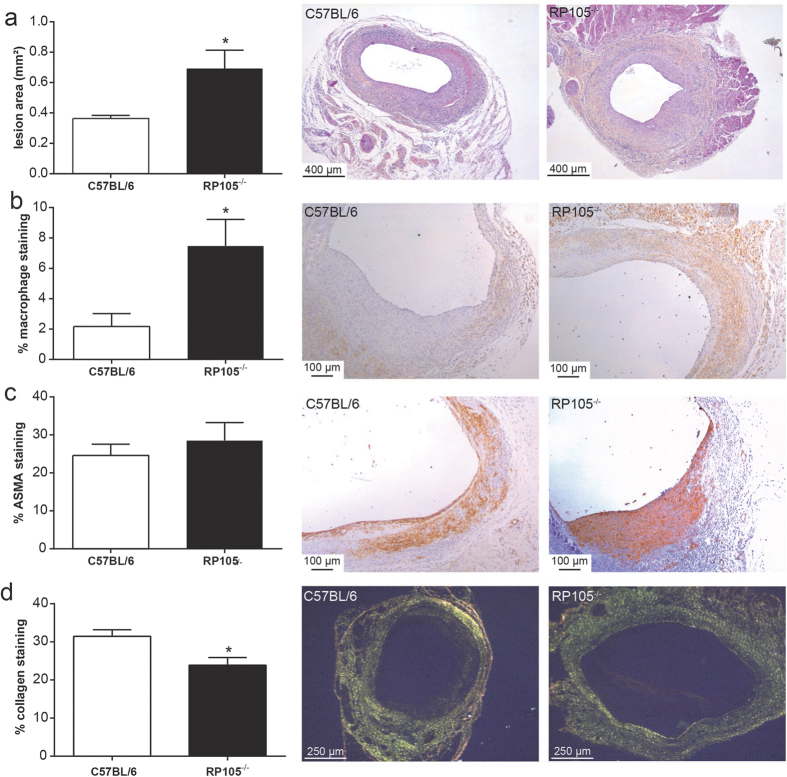
RP105 deficiency aggravates vein graft disease. Vein graft lesion area was significantly increased in RP105^−/−^ mice compared to control C57BL/6 mice (**a**). The macrophage content, expressed as the percentage of stained area in the intimal hyperplasia, was higher in RP105^−/−^ mice compared to control (**b**), while no changes were found in the percentage of smooth muscle cells (ASMA, alpha-smooth muscle cell actin, (**c**). Collagen content was decreased in RP105^−/−^ mice, indicative of less stable lesions (**d**). The micrographs show representative images of each group (50x). N = 11 C57BL/6 mice/group, N = 13 RP105^−/−^ mice/group. *P < 0.05.

Vein grafts were placed in RP105^−/−^ mice and control C57BL/6 mice to investigate the effect of RP105 deficiency on vein graft disease. Interestingly, lack of RP105 resulted in a 90% increased vein graft lesion area (Fig. 1a; P = 0.011). Plaque morphology was further analysed by staining for macrophages, SMC and collagen. The percentage of lesional macrophages was significantly increased in mice deficient for RP105 (Fig. 1b; P = 0.010), with a slight increase in both the relative M1 and M2 macrophage content as demonstrated by a MAC/3iNOS and a MAC3/CD206 staining (Supplemental Figure 2). No changes were observed in the percentage of SMC between the two groups (Fig. 1c). Picrosirius red staining revealed a significant reduction of collagen content in the lesions of RP105^−/−^ mice compared to control mice (Fig. 1d; P = 0.039), which indicates that the lesion may be more unstable. The absolute area of the individual staining are given in Supplemental Figure 3.

To further determine lesion stability, we analysed for plaque dissections. Dissections were defined as a fissure or connection between the lumen and part of the vessel wall, accompanied by the presence of erythrocytes and fibrin layers in the lesions. In RP105^−/−^ mice, 3 out of 13 mice displayed plaque dissections, while 0 out of 11 disruptions were observed in C57BL/6 mice.

### LDLr^−/−^/RP105^−/−^ mice display lesions with an unstable phenotype

**Figure 2:**
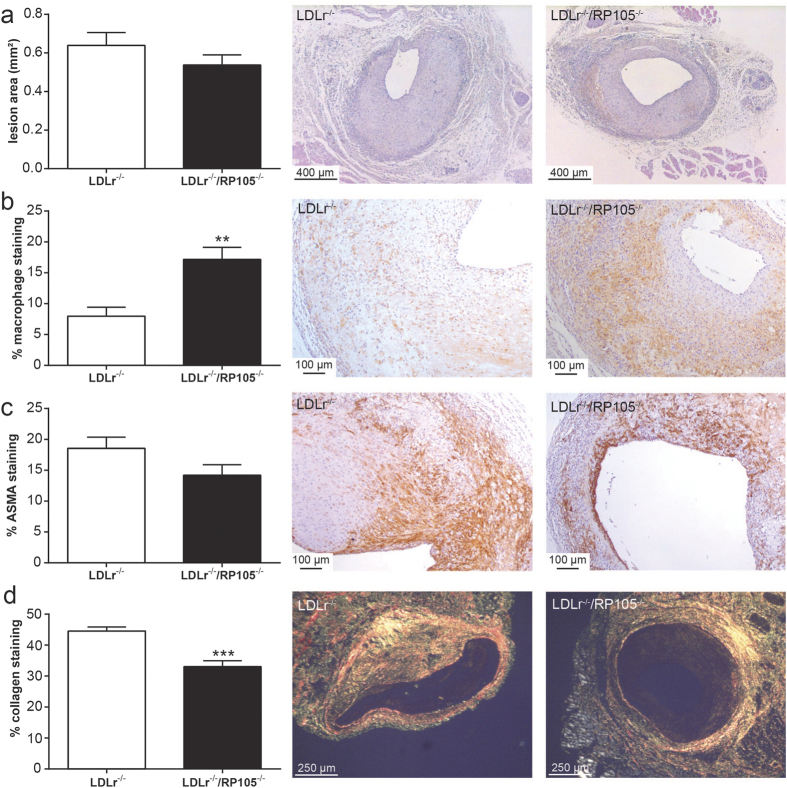
Lesion size and plaque morphology in LDLr^−/−^/RP105^−/−^ mice on a western type diet. No changes were observed in the vessel wall area of LDLr^−/−^/RP105^−/−^ mice compared to control LDLr^−/−^ mice (**a**). Similar to mice fed a chow diet, a higher percentage of macrophages was observed in LDLr^−/−^/RP105^−/−^ (**b**). Smooth muscle cell content was unaltered between the two groups (ASMA, (**c**)) and again a decrease in lesional collagen was observed (**d**). N = 12 LDLr^−/−^ mice/group. N = 12 LDLr^−/−^/RP105^−/−^ mice/group. *P < 0.05, **P < 0.01.

Next, we investigated whether high-fat diet feeding would alter the effects of RP105 deficiency on vein graft disease. LDLr^−/−^/RP105^−/−^ mice and LDLr^−/−^ mice as controls received autologous vein grafts while fed a western-type diet for 4 weeks. No changes were detected in total body weight (LDLr^−/−^: 27.7 ± 0.7 g versus LDLr^−/−^/RP105^−/−^: 28.2 ± 0.5 g, P = 0.38) or plasma total cholesterol levels (LDLr^−/−^: 1228 ± 68 mg/dL versus LDLr^−/−^/RP105^−/−^ mice: 1235 ± 82 mg/dL, P = 0.95) between the two groups. Furthermore, we did not observe significant differences between plasma VLDL/LDL cholesterol or HDL cholesterol (Supplementary Figure 4). In contrast to mice on chow diet, no changes in vein graft lesion area were observed in LDLr^−/−^/RP105^−/−^ mice compared to control LDLr^−/−^ mice (Fig. 2a; P = 0.173). Analysis of plaque morphology showed an increase in relative macrophage staining in lesions of LDLr^−/−^/RP105^−/−^ mice compared to control (Fig. 2b; P = 0.002). Relative M1 macrophage content did not differ between the groups, but we observed an increase in the relative amount of CD206^+^ macrophages in the LDLr^−/−^/RP105^−/−^ lesions compared to control (Supplemental Figure 5). We did not observe a significant difference in SMC staining between LDLr^−/−^/RP105^−/−^ mice and LDLr^−/−^ controls (P = 0.069; Fig. 2c). Relative lesional collagen content was significantly reduced in RP105 mice (Fig. 2d; P = 0.039). Absolute staining areas are displayed in Supplemental Figure 6.

**Figure 3:**
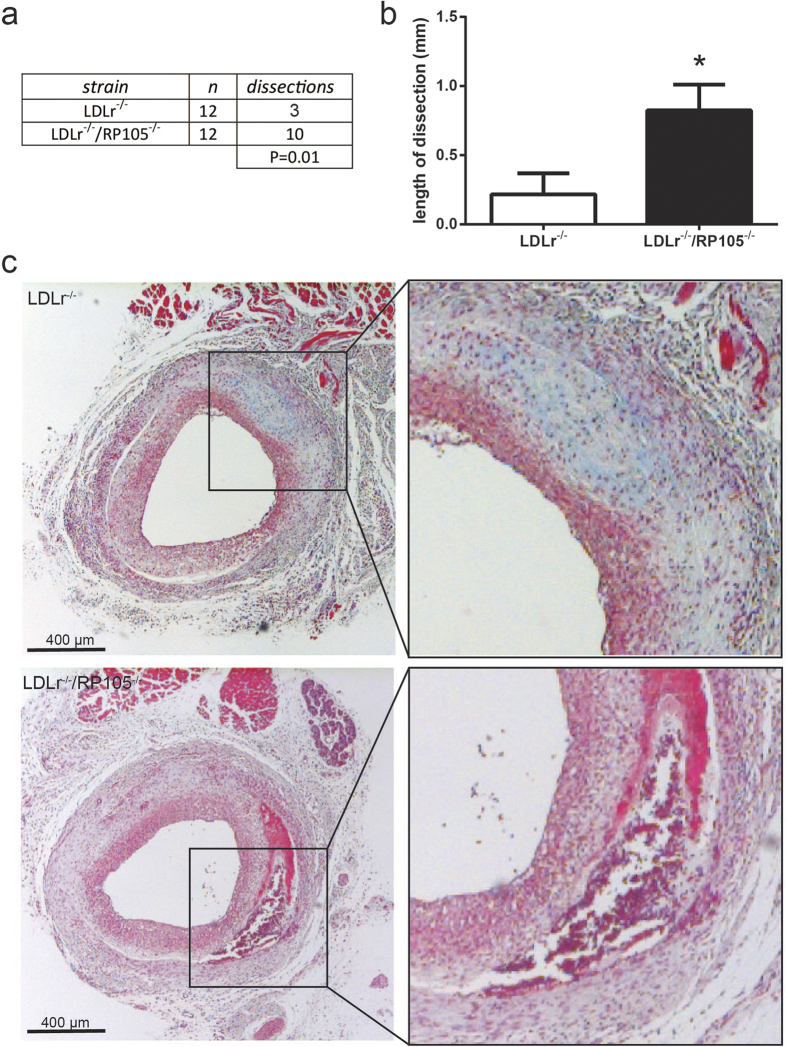
Increased plaque dissections in LDLr^−/−^/RP105^−/−^ mice. The number of plaque dissections in LDLr^−/−^/RP105^−/−^ mice was significantly higher compared to control LDLr^−/−^ mice (**a**). Also, the total length of plaque dissections was increased in LDLr^−/−^/RP105^−/−^ mice compared to control (**b**). The micrographs show representative images of each group 50x. N = 12 LDLr^−/−^ mice/group. N = 12 LDLr^−/−^/RP105^−/−^ mice/group. *P < 0.05.

The total number of plaque dissections and intraplaque haemorrhages was profoundly increased in LDLr^−/−^/RP105^−/−^ mice compared to LDLr^−/−^ control mice (LDLr^−/−^ mice: 3 out of 12 mice; LDLr^−/−^/RP105^−/−^: 10 out of 12 mice; Fig. 3a; P = 0.012, Fisher’s exact test). Furthermore, the average length of the dissections was higher in the LDLr^−/−^/RP105^−/−^ mice (Fig. 3b; P = 0.008).

### Unaltered MMP expression in RP105 deficient macrophages

As we observed a reduced collagen content in the lesions of RP105 deficient mice, and matrix metalloproteinases (MMPs) are known for its involvement in collagen homeostasis, we measured MMP expression in macrophages. However, no major changes were found in the expression of MMP2, MMP8 and MMP9 as well as in the expression of TIMP1, TIMP2 and TIMP3 at baseline or after stimulation with a concentration range of LPS in RP105^−/−^ macrophages compared to control (Supplemental Figure 7).

### Macrophage proliferation *in vitro* and *in vivo*

**Figure 4:**
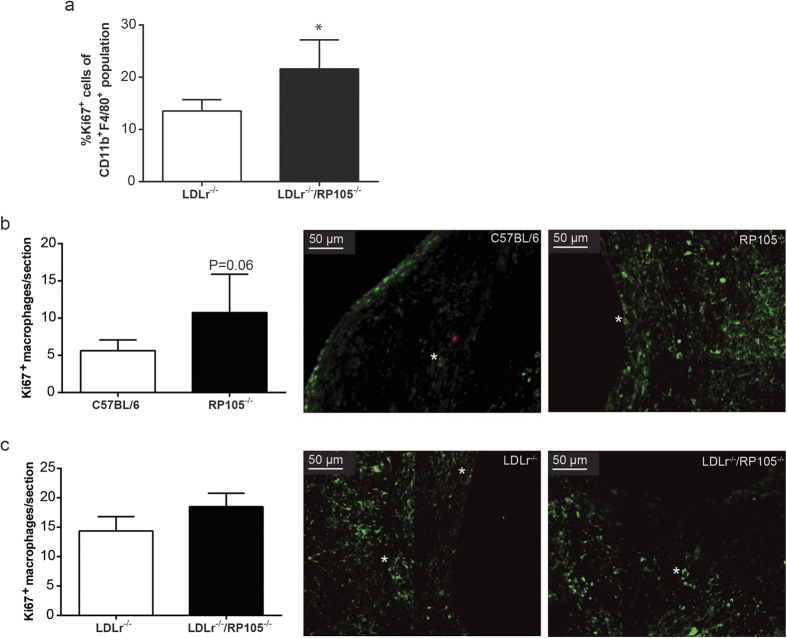
Macrophage proliferation. Peritoneal macrophages isolated from LDLr^−/−^/RP105^−/−^ mice showed an increased expression of the cellular proliferation marker Ki67 compared to peritoneal macrophages isolated from LDLr^−/−^ mice (**a)**, *P < 0.05). Intraplaque macrophage proliferation tended to be increased in RP105^−/−^ compared to C57Bl/6 mice (P = 0.06) as demonstrated by a MAC3/Ki67 double staining (**b**) (right micrographs, N = 11 C57BL/6 mice/group, N = 13 RP105^−/−^ mice/group). No significant differences could be detected in lesional macrophage proliferation between LDLr^−/−^ and LDLr^−/−^/RP105^−/−^ mice (**c**). N = 12 LDLr^−/−^ mice/group. N = 12 LDLr^−/−^/RP105^−/−^ mice/group.

Monocyte migration was recently established to be hampered upon RP105 deficiency in an atherosclerotic setting^17^. To elucidate the mechanisms behind the increased percentage of lesional macrophages observed in both *in vivo* studies, we thus aimed to determine macrophage proliferation, since it has recently been described that local macrophage proliferation may add to the lesional burden^18^. Peritoneal macrophages isolated from LDLr^−/−^/RP105^−/−^ mice, which are under the influence of a variety of proliferative stimuli *in vivo*, showed an increased expression of the cellular proliferation marker Ki67 compared to peritoneal macrophages isolated from LDLr^−/−^ mice (Fig. 4a). Also in the vein graft lesions of RP105^−/−^ mice, the number of Ki67^+^ macrophages (as illustrated in Fig. 4b) tended to be increased as compared to control vein grafts (P = 0.06, Fig. 4c). Although less pronounced, macrophage proliferation also seemed enhanced in the LDLr^−/−^/RP105^−/−^ lesions as compared to LDLr^−/−^ lesions (Fig. 4e). Taken together, these data demonstrate that RP105 deficient macrophages, under inflammatory conditions, are able to proliferate at a higher rate as compared to control macrophages.

### RP105^−/−^ SMC produce increased levels of CCL2

**Figure 5:**
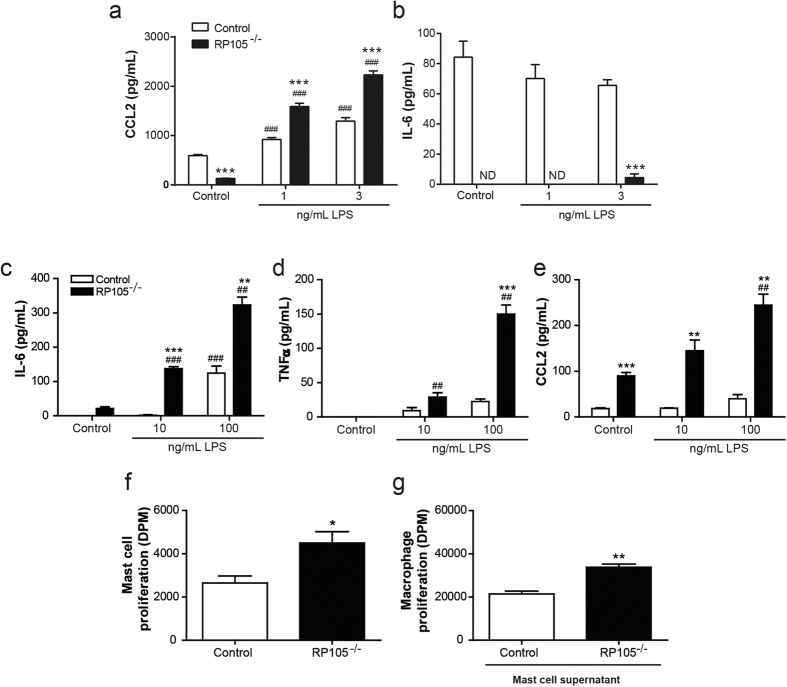
Effect of RP105 deficiency on smooth muscle cell and mast cell function. After 24 hours stimulation with 1 and 3 ng/mL LPS, RP105^−/−^ vascular smooth muscle cells (v SMCs) secrete dose-dependent increased levels of CCL2 (**a**), which was significantly higher compared to control v SMCs (N = 4). IL-6 secretion was significantly reduced in RP105^−/−^ v SMCs (**b**). ***P < 0.001 compared to control v SMCs. ^###^P < 0.001 compared to unstimulated v SMCs, nd =  not detectable. After 4 hours stimulation with 10 ng/mL and 100 ng/mL LPS, bone marrow derived RP105^−/−^ mast cells secreted dose-dependently increased levels of IL6 (**c**), TNFα (**d**) and CCL2 (**e**), which was significantly higher compared to control mast cells (N = 3, typical example of multiple experiments). The basal proliferation rate of RP105^−/−^ mast cells was significantly higher compared to control mast cells (**f**) (N = 4). Addition of the supernatant from RP105^−/−^ mast cells to macrophages resulted in increased proliferation compared to the addition of supernatant from control mast cells (**g**) (N = 4). *P < 0.05. ***P < 0.001 compared to control mast cells. ^#^P < 0.05, ^###^P < 0.001 compared to unstimulated mast cells.

To further examine why we observe the increased percentage of lesional macrophages in RP105 deficient mice, we determined CCL2 secretion *in vitro*, which is known to be one of the key chemokines involved in monocyte recruitment to the lesion^19^. Since the SMC is one of the major determinants of vein graft disease, we investigated whether SMC deficient in RP105 produce altered levels of CCL2. Indeed, primary cultured SMC lacking RP105 were seen to secrete significantly increased CCL2 amounts after stimulation with LPS, compared to control SMC (Fig. 5a; P = 0.0001). IL-6 secretion from vSMCs lacking RP105 was reduced as compared to control v SMCs (Fig. 5b; P = 0.0005), while TNFα secretion was not detectable under these conditions. Of note, RP105 deficiency did not alter release of CCL2, IL-6 or TNFα from macrophages (Supplemental Figure 8).

### RP105 deficiency aggravates mast cell activation

In addition to the SMC, we have previously shown that mast cells also play a key role in vein graft disease^20^. Moreover, mast cells can contribute to plaque destabilization, for instance via the degradation of collagen by mast cell derived tryptase and chymase^21^,^22^. Therefore, we aimed to determine the relevance of RP105 in mast cell function. First, we analysed whether cultured mast cells express RP105, which is indeed the case at both mRNA and protein level (Supplemental Figure 9). Next, we examined whether RP105 plays a functional role in mast cell activation by culturing bone marrow derived mast cells from RP105 deficient mice and control mice. Mast cells were stimulated with 10 ng/mL and 100 ng/mL LPS for 4 and 24 hours. RP105 deficient mast cells were seen to secrete higher levels of IL-6 and TNFα compared to control mast cells, which was dose-dependently increased after 4 hours of LPS activation (Fig. 5c,d). Also, CCL2 secretion of RP105^−/−^ mast cells was highly increased compared to control mast cells, which may also have contributed to the increased lesional macrophages *in vivo* (Fig. 5e). Furthermore, 24 hours of activation still showed a significant increase in IL-6 and CCL2 production of RP105^−/−^ mast cells compared to control. These data indicate that RP105 deficiency on the mast cell results in increased activation and cytokine release.

We then investigated whether cellular proliferation is altered in RP105 deficient mast cells. Interestingly, RP105 deficient mast cells show an increased proliferation rate compared to control mast cells under basal conditions (Fig. 5f).

Since we observed an increased activation status of RP105^−/−^ mast cells, and mast cells are known to secrete a variety of growth factors, we assessed whether the releasate of activated RP105^−/−^ mast cells has differential effects on macrophages compared to control mast cells. Indeed, after adding supernatant of RP105^−/−^ mast cells to macrophages, we observed an increased proliferation of macrophages compared to the addition of supernatant from control mast cells (Fig. 5g).

### Increased perivascular mast cell numbers and activation status *in vivo*

**Figure 6:**
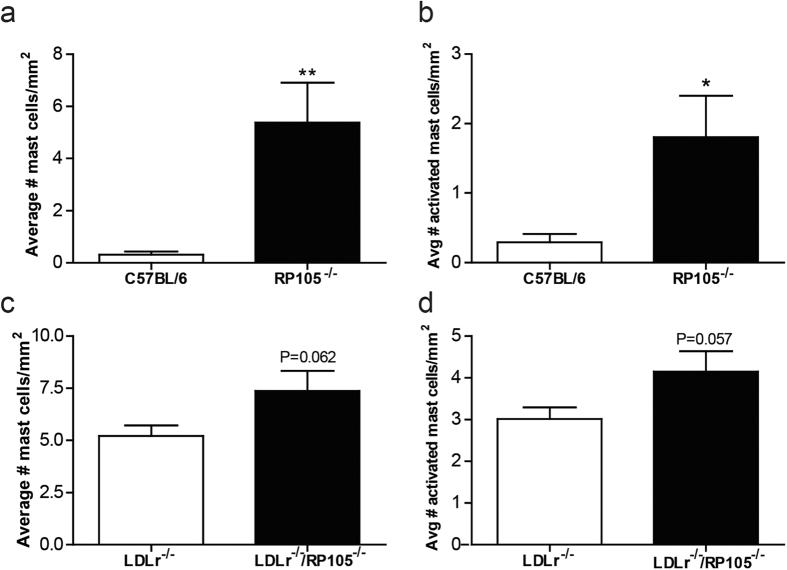
*In vivo* mast cell activation. The amount of perivascular mast cells was markedly increased in the perivascular tissue from vein grafts of RP105 deficient mice compared to control C57BL/6 mice (**a**). Also, the number of activated mast cells was significantly higher in RP105^−/−^ mice (**b**). Mast cell numbers, as well as the amount of activated mast cells, showed a trend towards an increase in LDLr^−/−^/RP105^−/−^ mice fed a western type diet compared to LDLr^−/−^ mice (**c,d**). N = 11 C57BL/6 mice/group, N = 13 RP105^−/−^ mice/group, N = 12 LDLr^−/−^ mice/group. N = 12 LDLr^−/−^/RP105^−/−^ mice/group. (1000x). *P < 0.05. **P < 0.01.

Considering the profound effects of RP105 deficiency on the mast cell found *in vitro*, we aimed to determine whether mast cell numbers and activation status *in vivo* are altered as well. Indeed, the average number of peri-adventitial mast cells was increased in RP105^−/−^ mice compared to control mice (Fig. 6a; P = 0.009). Moreover, in RP105^−/−^ mice the average number of activated mast cells was significantly higher (Fig. 6b; P = 0.033, Supplemental Figure 10).

In LDLr^−/−^ mice, basal mast cells levels were increased already upon western type diet feeding compared to C57BL/6 controls, which was further increased in mice lacking RP105, however this did not reach significance (Fig. 6c; P = 0.062). Also, the number of activated mast cells showed a trend towards an increase in LDLr^−/−^/RP105^−/−^ mice compared to control (Fig. 6d; P = 0.057).

### Increased CCL2 expression *in vivo*

**Figure 7:**
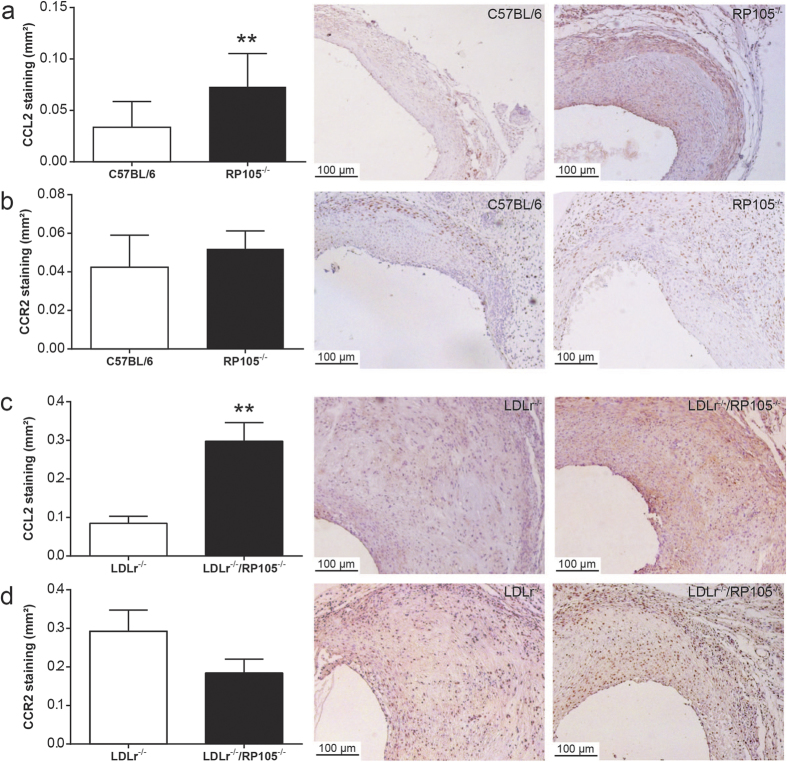
*In vivo* CCL2 and CCR2 staining. Immunohistochemical stainings revealed a significant increase in the CCL2 positive area in the lesions of RP105^−/−^ mice compared to control C57BL/6 mice (**a**). Also, the lesional CCL2 area was profoundly increased in hypercholesterolemic LDLr^−/−^/RP105^−/−^ mice compared to control LDLr^−/−^ mice (**c**). No differences were observed in lesional CCR2 expression between both experimental groups (**b,d**). N = 11 C57BL/6 mice/group, N = 13 RP105^−/−^ mice/group, N = 12 LDLr^−/−^ mice/group. N = 12 LDLr^−/−^/RP105^−/−^ mice/group. **P < 0.01.

Taking into consideration the increased amount of CCL2 secreted *in vitro* by both RP105 deficient SMC and mast cells, we aimed to determine whether lesional CCL2 expression was increased in RP105^−/−^ mice as well. Intriguingly, the CCL2 positive area in the lesions was significantly increased in RP105^−/−^ mice compared to control mice (Fig. 7a; P = 0.002). Also, the lesional CCL2 positive area in hypercholesterolemic LDLr^−/−^/RP105^−/−^ mice was profoundly increased compared to control mice (Fig. 7b; P = 0.0001). The higher expression of CCL2 in vein graft lesions of RP105 deficient mice may thus explain the increased macrophage content observed in these lesions. The expression of lesional CCR2, the receptor for CCL2 remained unaltered in both experimental groups (Fig. 7c,d).

## Discussion

In the current study we demonstrate that lack of the regulatory molecule RP105 results in a marked increase in vein graft lesion area. Furthermore, we show that in a hypercholesterolemic setting, RP105^−/−^ lesions display an increase in plaque dissections and plaque destabilization.

In our study, when comparing the C57Bl/6 and LDLr^−/−^ control groups, we observed an increase in lesion development upon high cholesterol feeding, which was, as described previously, caused by enhanced macrophage infiltration and collagen content, but also of lipid deposition in the plaque^13^. In a normocholesterolemic setup, RP105 deficiency resulted in an increase in vein graft disease mainly due to an increase in macrophage content, while lesion growth was similar between control and RP105 deficient mice under hypercholesterolemic conditions. We therefore postulate that RP105 deficiency in LDLr^−/−^ mice does not have an additive effect on lesion growth, because that is already highly induced by the increase in plasma cholesterol. Despite the lack of effects on lesion size in that study, we observed prominent effects of RP105 deficiency on lesion morphology as demonstrated by an increase in macrophage content, illustrative of an enhanced inflammatory response, and a decrease in collagen content, which gives rise to the stability of the lesion. More evident even was the increase in the number of plaque dissections, which is, as previously described^12^, a prominent marker for plaque instability in this vein graft model. Taken together, we thus conclude that RP105 deficiency in hypercholesterolemia results in reduced plaque stability as compared to the controls. In fact, in both studies decreased lesional collagen content was observed, while the macrophage content was enhanced. Our *in vitro* data demonstrate that RP105 deficient SMC as well as RP105^−/−^ mast cells secrete excessive levels of CCL2 compared to control cells, which may contribute to the increase of macrophages in the plaque. Also, local macrophage proliferation may be at least partly responsible for the increased macrophage content. Moreover, we are the first to demonstrate that RP105 plays a functional role in mast cell activation and proliferation, possibly aggravating lesion destabilization.

Mast cells are potent inflammatory cells which have previously been implicated in vein graft disease and plaque destabilization^20^,^21^,^22^,^23^. In those studies, mast cell deficiency led to reduced intimal hyperplasia, while activation of mast cells upon vein graft placement resulted in increased disease development^20^, thus establishing the importance of this cell type in vein graft disease. Mast cells exert their detrimental effects through the secretion of specific proteases and a variety of cytokines and chemokines, which all contribute to increased inflammation^24^. Activation of mast cells may be induced by a range of mediators such as IgE, neuropeptides^25^, complement factors^20^ or via toll like receptors, in particular TLR4^26^. Whether TLR4 signalling may actually lead to mast cell degranulation is still under debate, however, it is well established that TLR4 activation results in increased cytokine secretion^27^. RP105 is known to inhibit TLR4 signalling in macrophages and dendritic cells, however up to date evidence describing a role for RP105 in mast cells is lacking. Here, we have established that bone narrow derived mast cells express RP105 and as described previously, LPS dose-dependently increased the release of CCL2, IL-6 and TNFα by control mast cells. Interestingly, this increase was significantly higher in RP105 deficient mast cells, indicating that these cells, upon activation, secrete excessive amounts of cytokines in its surroundings. We now show that also on the mast cell, RP105 has an inhibitory effect and consequently, lack of RP105 leads to increased activation. Therefore, we postulate that the increase in vessel wall thickening observed in RP105^−/−^ mice on a chow diet may at least partly be caused by aggravated mast cell activation. Also, mast cell derived tryptase and chymase have previously been shown to increase collagen degradation^22^, which thus may have contributed to the decreased lesional collagen content observed in the current study.

In LDLr^−/−^/RP105^−/−^ mice fed a western type diet, we observed a decrease in collagen content as well, and an increase in plaque dissections, which was however accompanied by a less pronounced effect on mast cell activation. We have previously established that upon western type diet feeding, vascular mast cell numbers are enhanced^22^. Indeed, control LDLr^−/−^ mice fed a western type diet already displayed higher numbers of activated mast cells, compared to control C57BL/6 mice on a chow diet. Therefore, the additive inflammatory effect of RP105 deficiency on mast cells in mice receiving a high cholesterol diet may be less evident. Nevertheless, RP105 deficiency in a hypercholesterolemic setting resulted in a severe decrease in plaque stability, thus establishing a critical role for RP105 in maintaining vein graft integrity.

Previously, we have investigated the effects of RP105 in other mouse models of vascular remodelling as well. Besides vein graft surgery, balloon angioplasty with stent placement is used for the treatment of atherosclerosis; however in-stent restenosis impairs the success rate of this operation^28^. In a murine mouse model for restenosis we have shown that RP105 deficiency results in increased neo-intima formation^29^, which is in line with the observed effects on vein graft disease in the current study. In that study, overexpression of soluble RP105 in complex with MD1 inhibited neointima formation, while single RP105 or MD1 were ineffective, suggesting that the soluble RP105-MD1 complex is therapeutically relevant for vein graft disease as well. Conversely, in a setting of atherosclerosis, mice on a western type diet lacking RP105 on their myeloid cells develop reduced atherosclerotic lesions due to changes in B cells^30^. Also, total body RP105 deficiency was seen to reduce atherosclerosis and decrease lesional macrophage content, caused by a reduced monocyte influx^17^. These disturbances in monocyte migration were also observed in RP105^−/−^ mice with hind limb ischemia^31^. The fact that we observe an increase in lesional macrophages may be explained by the profound increase in CCL2 secretion by both RP105^−/−^ SMC and mast cells, which could have resulted increased monocyte recruitment to the lesion *in vivo*. As vein graft lesions are highly enriched in SMC compared to atherosclerotic plaques, the effect of SMC derived products, such as CCL2, may be more prominent in vein graft disease as compared to atherosclerosis. We have previously shown that the CCR2-CCL2 axis is of vital importance for the development of vein graft disease, as inhibition of this specific chemokine receptor-ligand interaction, by either a specific antagonist^32^ or a shRNA targeting CCR2^33^, effectively inhibited intimal hyperplasia. In this study, CCL2 expression in the vein graft lesions of mice deficient for RP105 was significantly increased compared to controls, which can, at least partly, explain the increase in macrophage content and plaque progression. Macrophage polarization did not seem to be largely affected *in vivo*, as we observed similar to slightly higher levels of both M1 and M2 macrophages in lesions of RP105 deficient animals. Under hyperlipidemic condition, M2 macrophage levels were somewhat increased, but in lesions of both the control and RP105 deficient mice the number of M1 macrophages highly exceeds the amount of M2 macrophages, rendering an anti-inflammatory and plaque resolving activity of these relatively few macrophages unlikely. Also *in vitro*, cytokine secretion did not differ between macrophages derived from control or RP105deficient mice, suggesting that RP105 does not directly affect macrophage polarization.

RP105 has also been implicated in the induction of apoptosis. Literature regarding RP105-mediated effects on apoptosis are however rather contradictory, and seem to largely depend on cell type investigated. It has for example been shown that RP105 protects against apoptosis in cardiomyocytes^34^, but it has also been established that an anti-RP105 antibody can rescue B cells from apoptosis^35^. We have previously established that the amount of apoptotic cells in vein graft lesions is very low (~2% of the total amount of cells)^36^, and therefore we regard it unlikely that a further reduction in the already low amount of apoptosis will lead to the observed increase in intraplaque macrophages upon RP105 deficiency. Finally, we observed that macrophages of RP105^−/−^ mice tend to have an increased proliferation rate as compared to control macrophages under inflammatory conditions. Macrophage proliferation in atherosclerotic plaques has been described previously^18^, and in our study this phenomenon may be another contributing mechanism to the observed increase in lesion macrophage content. The contrasting effects of RP105 seen in this and previous studies on spontaneous atherosclerosis highlight the difference in underlying mechanisms of diet-induced atherosclerotic lesion development versus restenosis and vein graft disease, the latter two in which smooth muscle cells play a dominant role.

In summary, the current study is the first to demonstrate that lack of RP105 results in increased lesion area in murine vein grafts. In a hypercholesterolemic setting, RP105 deficiency resulted in an increase in lesion destabilization and a higher number of plaque dissections accompanied by intraplaque hemorrhage. *In vitro* studies suggest that this may be caused by excessive CCL2 secretion by RP105^−/−^ SMC, increased recruitment and proliferation of RP105^−/−^ monocytes and macrophages, as well as by an increased inflammatory and proliferative phenotype of RP105^−/−^ mast cells.

## Material and methods

### Mice

All animal experiments were approved by the animal welfare committee of the Leiden University Medical Center (approval reference number 09098 and 12153). This study was performed in compliance with Dutch government guidelines and the Directive 2010/63/EU of the European Parliament. RP105^−/−^ mice were kindly provided by K. Miyake (Tokyo University, Japan) and were described previously^16^. Male wild type (WT, C57BL/6, 10–12 weeks old) (n = 11) and RP105^−/−^ animals (C57BL/6 background, backcrossed for more than 10 generations, 10–12 weeks old) (n = 13) were bred in our facility at the Leiden University Medical Center (Leiden, The Netherlands) and during the experiment, mice were given water and chow *ad libitum*.

To study the effects of RP105 deficiency on vein graft disease in a hyperlipidemic setting, male LDLr^−/−^ mice (n = 12, 12–16 weeks old) and male LDLr^−/−^/RP105^−/−^ mice (n = 12, 12–16 weeks old) were used. Mice were fed a western-type diet containing 0.25% cholesterol and 15% cacaobutter (SDS) for 4 weeks. Plasma total cholesterol levels were measured by enzymatic procedures using precipath standardized serum as an internal standard (Boehringer Mannheim). The distribution of cholesterol among the different lipoproteins was determined by loading 30 μL serum onto a Superose 6 column (3.2 × 30 mm; Smart-system, GE Healthcare). Serum was fractionated at a constant flow rate of 50 μL/min with PBS. Total cholesterol in the effluent were determined enzymatically.

### Surgical intervention

Before surgery and sacrifice, mice were anesthetized by an intra-peritoneal injection with Midazolam (5 mg/kg, Roche), Dexdomitor (0.5 mg/kg, AST Farma) and fentanyl (0.05 mg/kg, Janssen). Adequacy of anaesthesia was monitored by regular visual inspection and toe pinch reflex. After surgery mice were antagonized with a subcutaneous injection of flumazenil (0.5 mg/kg, Fresenius Kabi,) and Antisedan (2.5 mg/kg, AST farma). Buprenorphine (0.1 mg/kg, MSD animal Health) was given after surgery to relieve pain. Vein graft surgery was performed in order to induce intimal hyperplasia in the venous grafts^11^. In brief, the carotid artery of the recipient mice was cut in the middle; both ends of the artery were everted around cuffs and ligated with 8.0 sutures. Donor littermates were anesthetized as described above, after which the vena cava was harvested and donor mice were exsanguinated. The vena cava was then sleeved over the cuffs in the recipient mice. At sacrifice after 28 days, mice were exsanguinated via orbital bleeding. Vein grafts were harvested and fixated in 4% formaldehyde, dehydrated and paraffin-embedded for histology.

### Histological and immunohistochemical analysis

All (immuno)-histochemical stainings and measurements were performed on six consecutive cross-sections, approximately 150 μm interspaced, of paraffin embedded vein grafts segments (5 μm thick). Hematoxylin-phloxine saffron (HPS) staining and Masson trichrome stainings were used for the measurement of vein graft lesion area and plaque dissection analysis, while collagen content was visualized with a picrosirius red staining under polarized light. Composition of the vein graft lesions was further evaluated by staining for macrophages (MAC3, BD-Pharmingen 550292), smooth muscle cell actin (Sigma; 1A4), CCL2 ((Novus Biologicals, NBD1-42280) and CCR2 (Abcam; ab222324). M1 macrophages were identified using a MAC3/iNOS (iNOS, Novus Biologicals; NB300-605) double staining, whereas M2 macrophages were visualized with a MAC3/CD206 (Abcam; ab64693) double staining. Intraplaque macrophage proliferation was determined by staining sections for MAC3 and the proliferation marker Ki67 (Abcam; ab16667). Double stained slides were scanned with a digital slide scanner (Pannoramic 250 Flash, 3DHistech Ltd). Double positive MAC3/Ki67 cells were counted manually, whereas MAC3/iNOS (M1 macrophages) and MAC3/CD206 (M2 macrophages) positive cells were scored semi quantitative using the following system for each of the macrophages subset (0: no double staining, 1: <10% of macrophages were positive for the individual M1 or M2 marker, 2: >10% < 20% MAC3/iNOS+ or MAC3/CD206+ cells, 3: >20% MAC3/iNOS+ or MAC3/CD206+ cells). Mast cells were histochemically stained using an enzymatic chloroacetate esterase kit (Sigma-Aldrich) and their numbers were microscopically counted. A mast cell was considered resting when all granules were maintained inside the cell; when granules were apparent in the vicinity of the mast cell they were scored as activated. Plaque dissection analysis was performed over a total vein graft length of 1800 μm. Disruptions were defined as a connection or fissure between the lumen and part of the vessel wall underneath the adventitia, filled with fibrin and erythrocytes^11^. Quantification of the lesion area and immunostained positive area were performed using computer assisted software (Leica Qwin, Leica). In brief, the total intimal area was measured, as well as the stained area within the intimal area. The stained area was than calculated as a percentage of the total intimal area. Negative control stainings are given in Supplemental Figure 1, establishing the specificity of the used antibodies. All measurements were performed in a blinded manner by a single observer.

### Cell culture

LDLr^−/−^ and LDLr^−/−^/RP105^−/−^ mice were anaesthetized as described above and sacrificed via cervical dislocation, after which bone marrow (BM) suspensions were isolated by flushing the femurs and tibias with PBS. BM derived mast cells (BMMCs) were grown by culturing BM cells from LDLr^−/−^ mice and LDLr^−/−^/RP105^−/−^ mice at a density of 0.25*10^6^ cells in RPMI containing 10% fetal bovine serum (FBS), 2 mmol/L l-glutamine, 100 U/mL penicillin, 100 μg/mL streptomycin (all from PAA) and mIL3 supernatant (supernatant from WEHI cells overexpressing murine Interleukin (IL)-3) for 4 weeks. RAW 264.7 cells (a murine macrophage cell line) were cultured in Dulbecco’s modified Eagle’s medium (DMEM) supplemented with 10% Fetal Calf’s Serum (FCS), 2 mmol/L L-glutamine, 100 U/mL penicillin and 100ug/mL streptomycin.

To generate bone marrow derived macrophages (BMDM), cells from bone marrow of LDLr^−/−^ mice and LDLr^−/−^/RP105^−/−^ mice were cultured for 7 days in RPMI medium supplemented with 20% fetal calf serum (FCS), 2 mmol/L l-glutamine, 100 U/mL penicillin, 100 μg/mL streptomycin (all from PAA) and 30% L929 cell-conditioned medium (as the source of macrophage colony-stimulating factor (M-CSF)) in petridishes (Greiner Bio-one)^37^.

Murine v SMCs explanted from aortas of RP105^−/−^ mice and control mice (anesthetized as described above and sacrificed via asphyxiation) were cultured and incubated overnight with either LPS (1 ng/mL or 3 ng/mL) or control medium (n = 4) in 0.2% FBS. IL-6, TNFα and CCL2 ELISAs were performed according to manufacturer’s protocol (BD Biosciences).

### Macrophage activation

Bone marrow derived LDLr^−/−^ and LDLr^−/−^/RP105^−/−^ macrophages cultured as described above, were activated with 1 ng/mL, 10 ng/mL or 50 ng/mL LPS (from E. Coli, Sigma-Aldrich) for 4 hours (n = 3) at a density of 10^6^ cells per well. Cells were then spun down (1500 rpm, 5 minutes) and used for RNA isolation. Supernatant was used to measure cytokine secretion by ELISA according to manufacturer’s protocol.

### Proliferation assay

To measure the effect of RP105 on macrophage proliferation, LDLr^−/−^/RP105^−/−^ BMDM and control LDLr^−/−^ BMDM were seeded at a density of 4*10^4^ cells per well (n = 4). Cells were incubated overnight with 0.5 μCi [^3^H]thymidine (PerkinElmer) per well at 37 °C. [^3^H]Thymidine incorporation was quantified in a liquid scintillation analyser (Packard 1500 Tricarb,).

To investigate the effect of RP105 deficiency on mast cell proliferation, LDLr^−/−^ and LDLr^−/−^/RP105^−/−^ BMMCs were seeded at a density of 10^4^ cells per well. Cells were incubated overnight with 0.5 μCi [^3^H]thymidine (PerkinElmer) per well. [^3^H]Thymidine incorporation was measured as described above. The effect of RP105^−/−^ mast cell supernatant on macrophages proliferation was investigated by seeding RAW cells at a density of 4*10^4^ cells per well. The next day, cells were exposed to supernatant of non-activated LDLr^−/−^ or LDLr^−/−^/RP105^−/−^ mast cells (n = 4). After overnight incubation 0.5 μCi [^3^H]thymidine (PerkinElmer), proliferation was measured as described above.

To measure macrophage proliferation in an *in vivo* setting, peritoneal macrophages were isolated from LDLr^−/−^ and LDLr^−/−^/RP105^−/−^ mice (anaesthetized as described above and sacrificed via cervical dislocation) by lavage of the peritoneal cavity with PBS (10 mL). Expression of the surface markers CD11b (CD11b-PE, Ebioscience, 12-0112-82), F4/80 (F4/80-APC, Ebioscience, 17-4801-82) and the proliferation marker Ki67 (KI67-FITC, Ebioscience, 11-5698-82) was determined by means of FACS analysis (FACS Canto, BD Biosciences).

### Mast cell activation

LDLr^−/−^ and LDLr^−/−^/RP105^−/−^ BMMCs were seeded at a density of 10^6^ cells per well (n = 3), after which they were stimulated with 1 ng/mL, 10 ng/mL or 100 ng/mL LPS (from E. Coli, Sigma-Aldrich, St. Louis, MO, USA). After 4 hours and 24 hours activation, cells were spun down (1500 rpm, 5 minutes) and the supernatant was collected for further analysis. ELISAs were performed according to manufacturer’s protocol. Expression of the surface markers RP105 (RP105-PE, Ebioscience, 12-1801-81) and TLR4 (TLR4-APC, Ebioscience, 12-9924-82) on control LDLr^−/−^ mast cells was determined by means of FACS analysis.

### RNA isolation, cDNA synthesis and qPCR

Guanidine thiocyanate (GTC) was used to extract total RNA from BMMCs and BMDMs^38^. RNA was reverse transcribed by M-MuLV reverse transcriptase (RevertAid, MBI Fermentas) and used for quantitative analysis of mouse genes (Supplemental Table 1) with an ABI PRISM 7700 Taqman apparatus (Applied Biosystems). Murine hypoxanthine phosphoribosyltransferase (HPRT) and murine ribosomal protein 27 (RPL27) were used as standard housekeeping genes.

### Statistical analysis

All data are presented as mean ± SEM. A 2-tailed Student’s t-test was used to compare individual groups. When comparing more than 2 groups, a 2-way ANOVA was performed with a Bonferroni post-test. Non-Gaussian distributed data were analysed using a two-tailed Mann-Whitney U test. Frequency data analysis was performed by means of the Fisher’s exact test. A P-level of <0.05 was considered statistical significant.

## Additional Information

**How to cite this article**: Wezel, A. *et al*. Deficiency of the TLR4 analogue RP105 aggravates vein graft disease by inducing a pro-inflammatory response. *Sci. Rep.* **6**, 24248; doi: 10.1038/srep24248 (2016).

## Supplementary Material

Supplementary Information
