# Supplementary material for: Deficiency of the TLR4 analogue RP105 aggravates vein graft disease by inducing a pro-inflammatory response
Source: Sci Rep. 2016 Apr 7;6:24248. doi: 10.1038/srep24248 (PMC4823661; doi:10.1038/srep24248)

**Deficiency of the TLR4 analogue RP105 aggravates vein graft disease by inducing a pro-inflammatory response**

^#^Anouk Wezel^1,2^, ^#^*Margreet R. de Vries^2,3^, Johanna M. Maassen^1^, Peter Kip^2^, Erna A. Peters^2,3^, Jacco C. Karper^2,3^, Johan Kuiper^1^, Ilze Bot^1,2^, Paul H.A. Quax^2,3^.

**Supplemental data**

**Supplemental Table 1.** List of primers used for the *in vitro* experiments

| **Gene** | **Forward primer** | **Reversed primer** |
| --- | --- | --- |
| RP105 | ACCATTCAAAACACGACCTTCAGCAGA | GGGGATTTGCGGTTAGTACAAGTGTGT |
| TLR4 | CCAATTTTTCAGAACTTCAGTGGCTGG | TTGAGAGGTGGTGTAAGCCATGC |
| MMP2 | CCGAGGACTATGACCGGGATA | GGGCACCTTCTGAATTTCCA |
| MMP8 | TGACCTCAATTTCATATCTCTGTTCTG | TCATAGCCACTTAGAGCCCAGTACT |
| MMP9 | CCCTGGAACTCACACGACATCTTC | CTCATTTTGGAAACTCACACGCCAG |
| TIMP1 | ACACCCCAGTCATGGAAAGC | CTTAGGCGGCCCGTGAT |
| TIMP2 | GTTTATCTACACGGCCCCCTCTT | ATCTTGCCATCTCCTTCTGCCTT |
| TIMP3 | ACTGTGCAACTTTGTGGAGAGGT | GAGACACTCATTCTTGGAGGTCA |
| HPRT | TTGCTCGAGATGTCATGAAGGA | AGCAGGTCAGCAAAGAACTTATAG |
| β-actin | CGCCAAGCGATCCAAGATCAAGTCC | AGCTGGGTCCCTGAACACATCCTTG |

**Supplemental Figure 1.** Isotype controls of each individual staining.


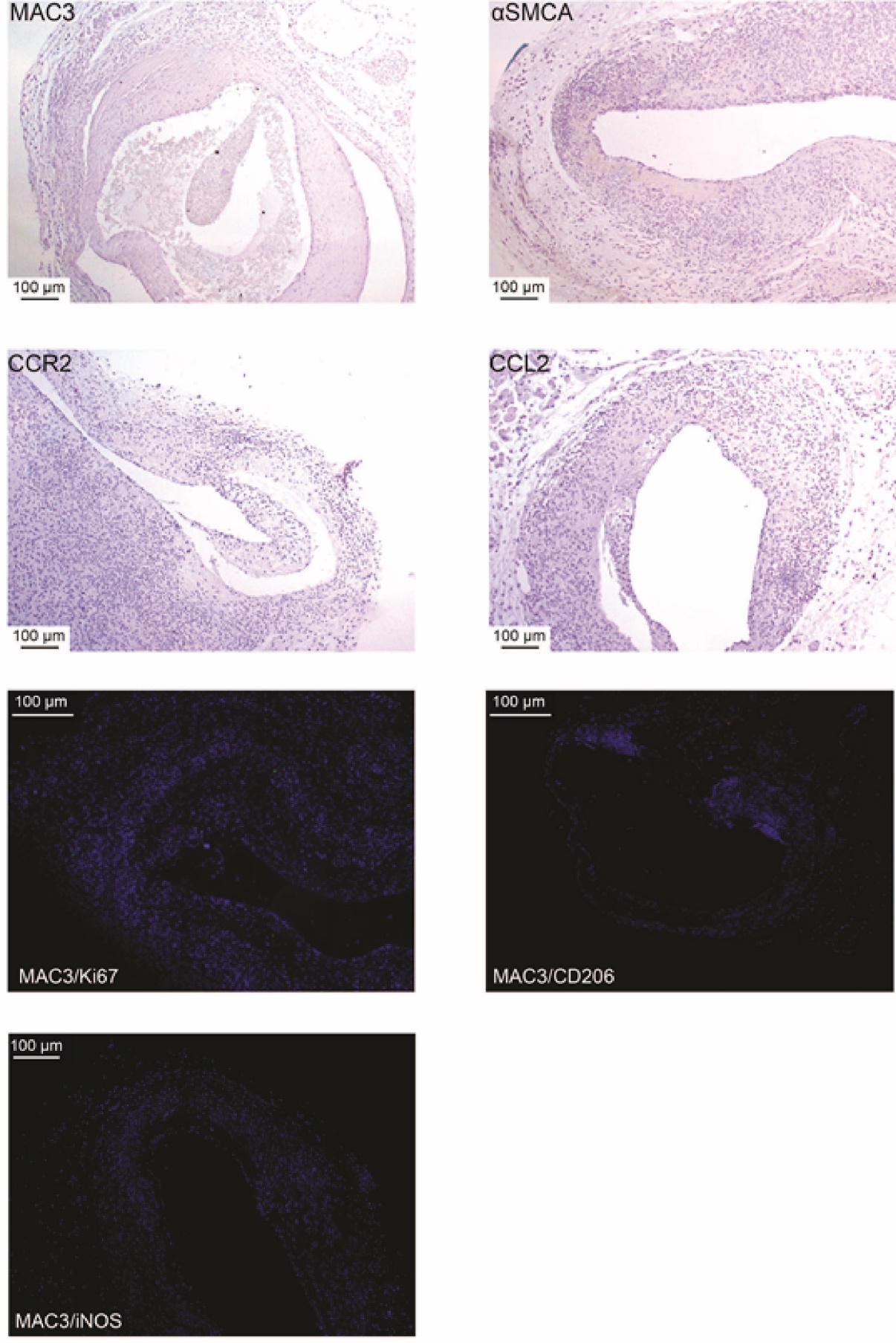


**Supplemental Figure 2.** M1 (A) and M2 (B) macrophage score in lesions of C57Bl6 controls versus RP105^-/-^ mice as determined by a MAC3/iNOS (M1) and a MAC3/CD206 (M2) double staining. In the micrographs * indicates double positive cells. The white line indicates the border between the media and adventitia. M1 and M2 macrophage score did not significantly differ between the C57Bl/6 and RP105^-/-^ mice (P=0.08 and P=0.15, respectively).

**
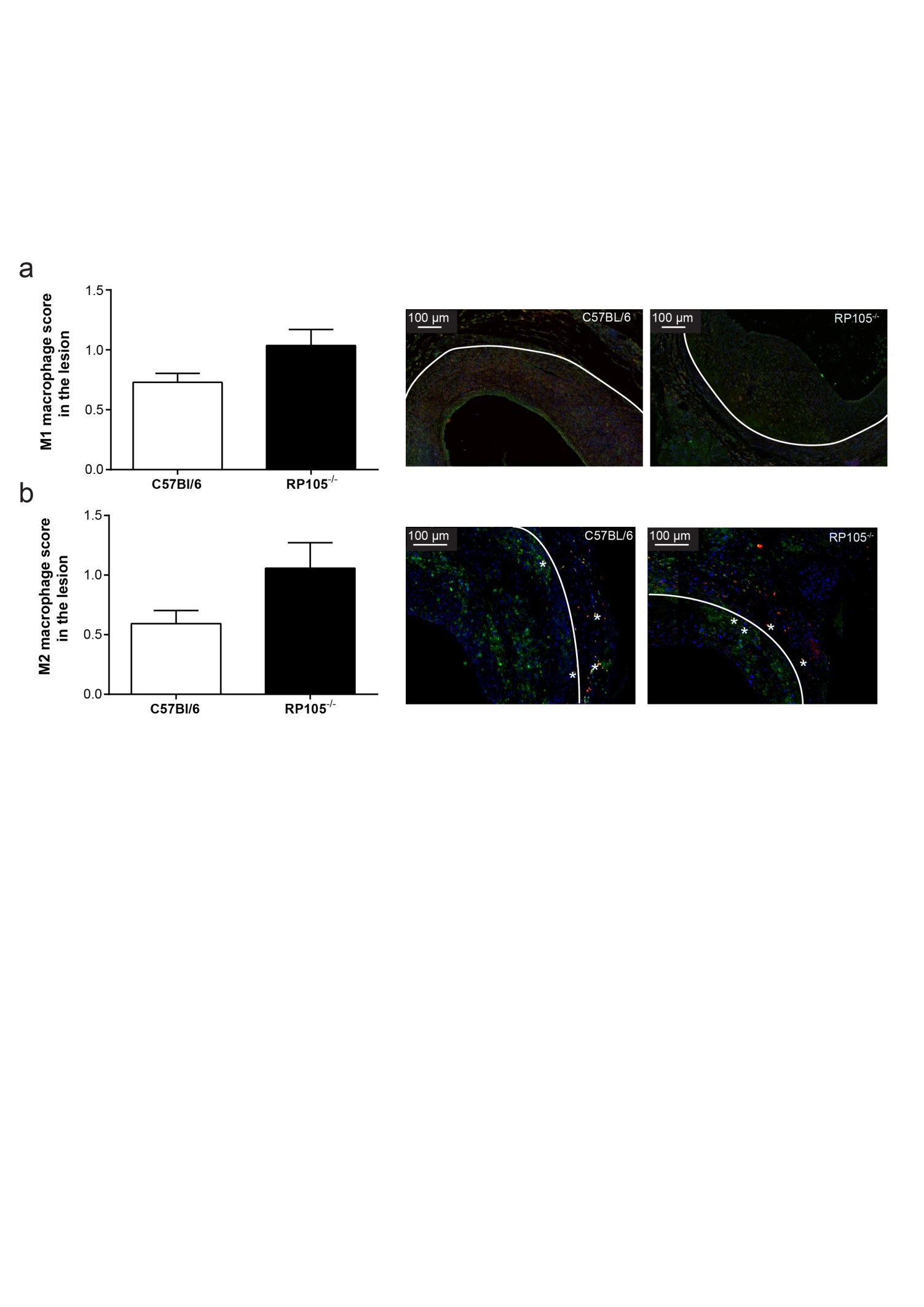
**

**Supplemental Figure 3.** Absolute area of the A) macrophage, B) smooth muscle cell (ASMA) and C) collagen staining in C57Bl/6 versus RP105^-/-^ mice. *P=0.02.

**
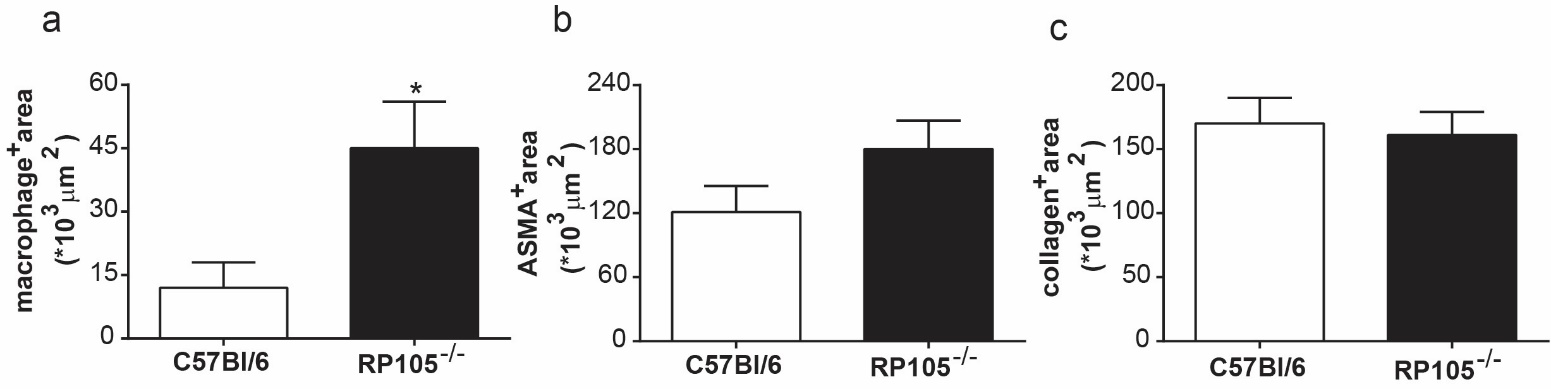
**

**Supplemental Figure 4.** Plasma lipoprotein fractions of LDLr^-/-^ versus LDLr^-/-^/RP105^-/-^ mice.

**
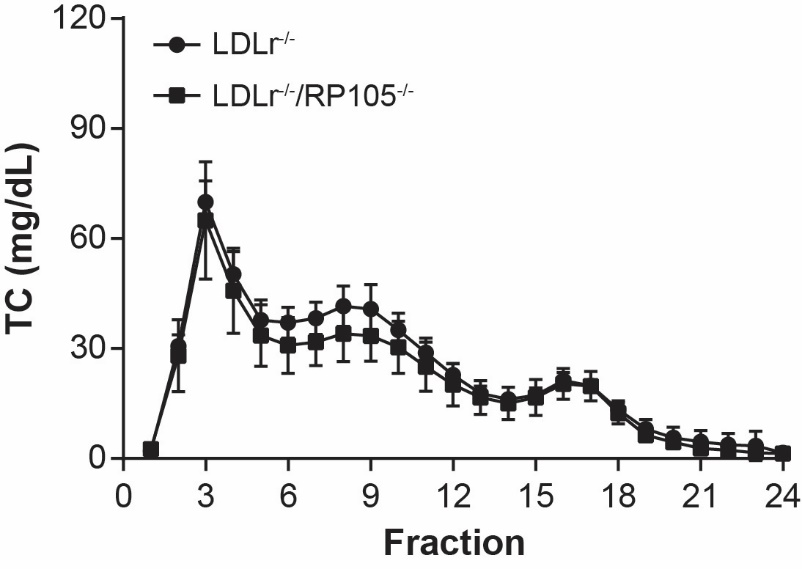
**

**Supplemental Figure 5.** M1 (a) and M2 (b) macrophage score in lesions of LDLr^-/-^ controls versus LDLr^-/-^/RP105^-/-^ mice as determined by a MAC3/iNOS (M1) and a MAC3/CD206 (M2) double staining. In the micrographs * indicates double positive cells. The white line indicates the border between the media and adventitia.

**
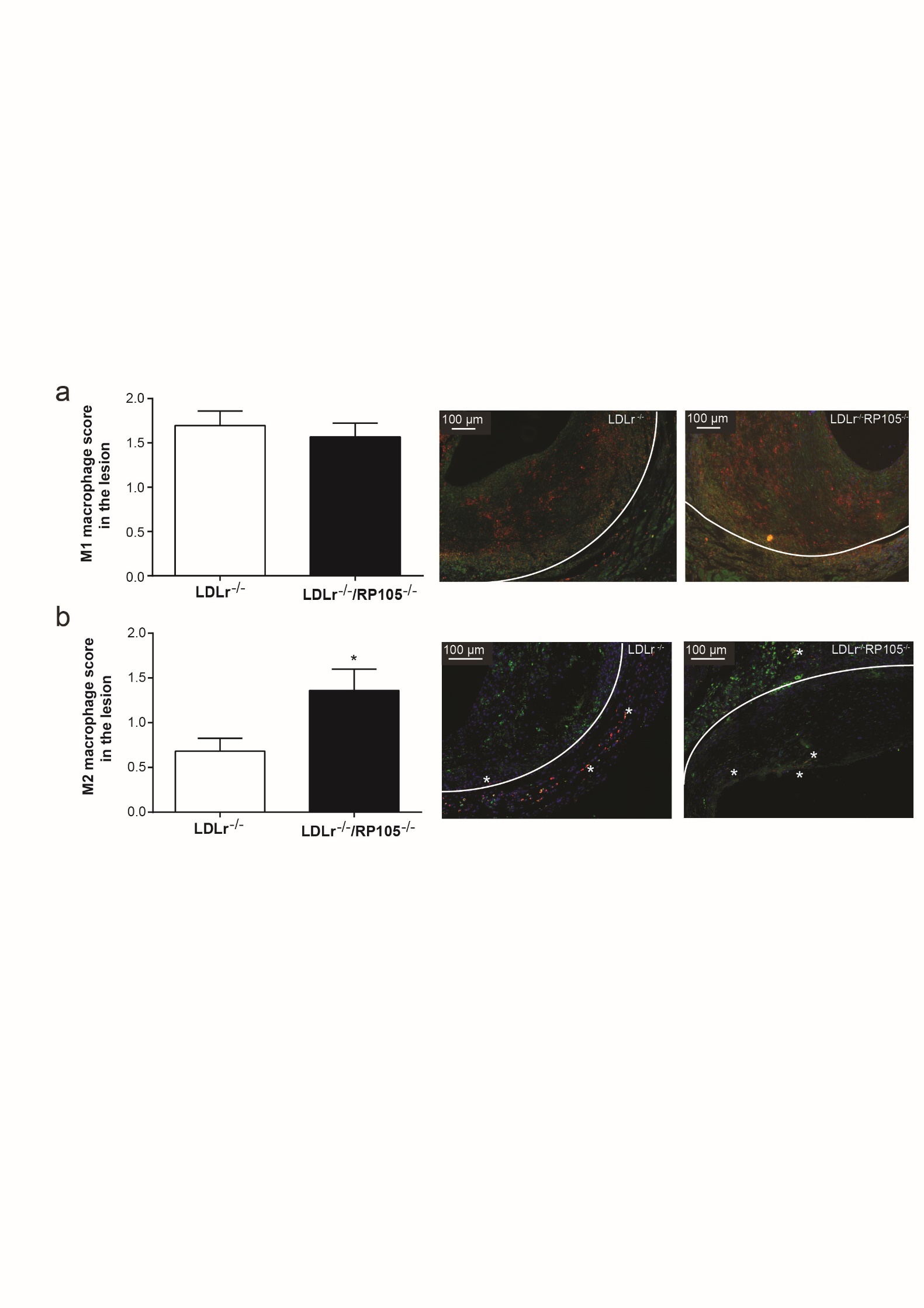
**

**Supplemental Figure 6.** Absolute area of A) macrophage, B) smooth muscle cell (ASMA) and C) collagen staining in LDLr^-/-^ versus LDLr^-/-^/RP105^-/-^ mice. *P=0.003.


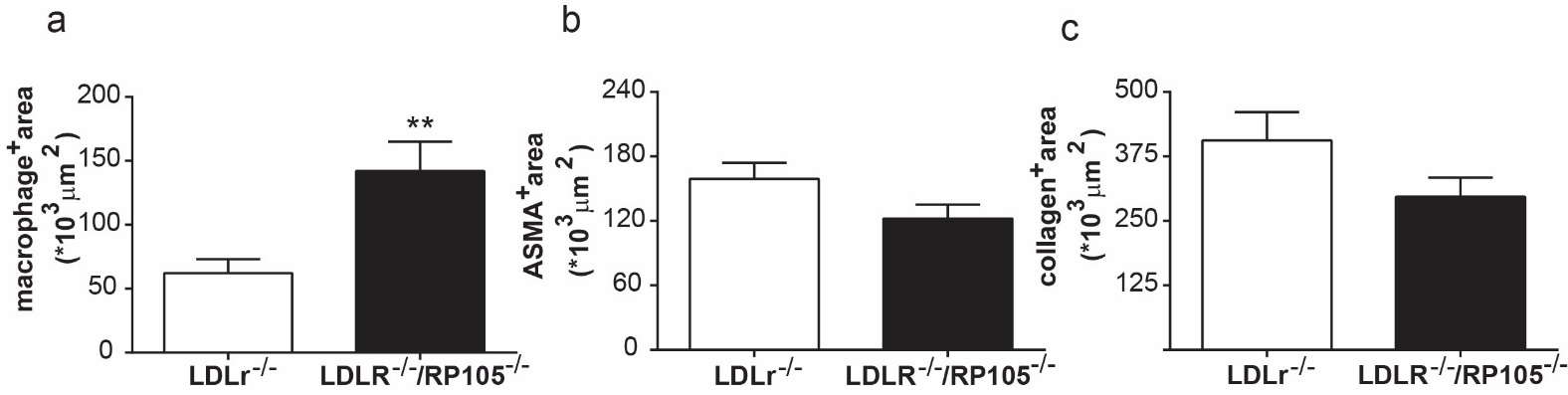


**Supplemental Figure 7.** Relative expression of MMPs and TIMPs in RP105 deficient and control BM derived macrophages after stimulation with LPS, or with control medium. *P=0.036 (MMP2) and *P=0.014 (MMP9), **P=0.0036 compared to control macrophages.


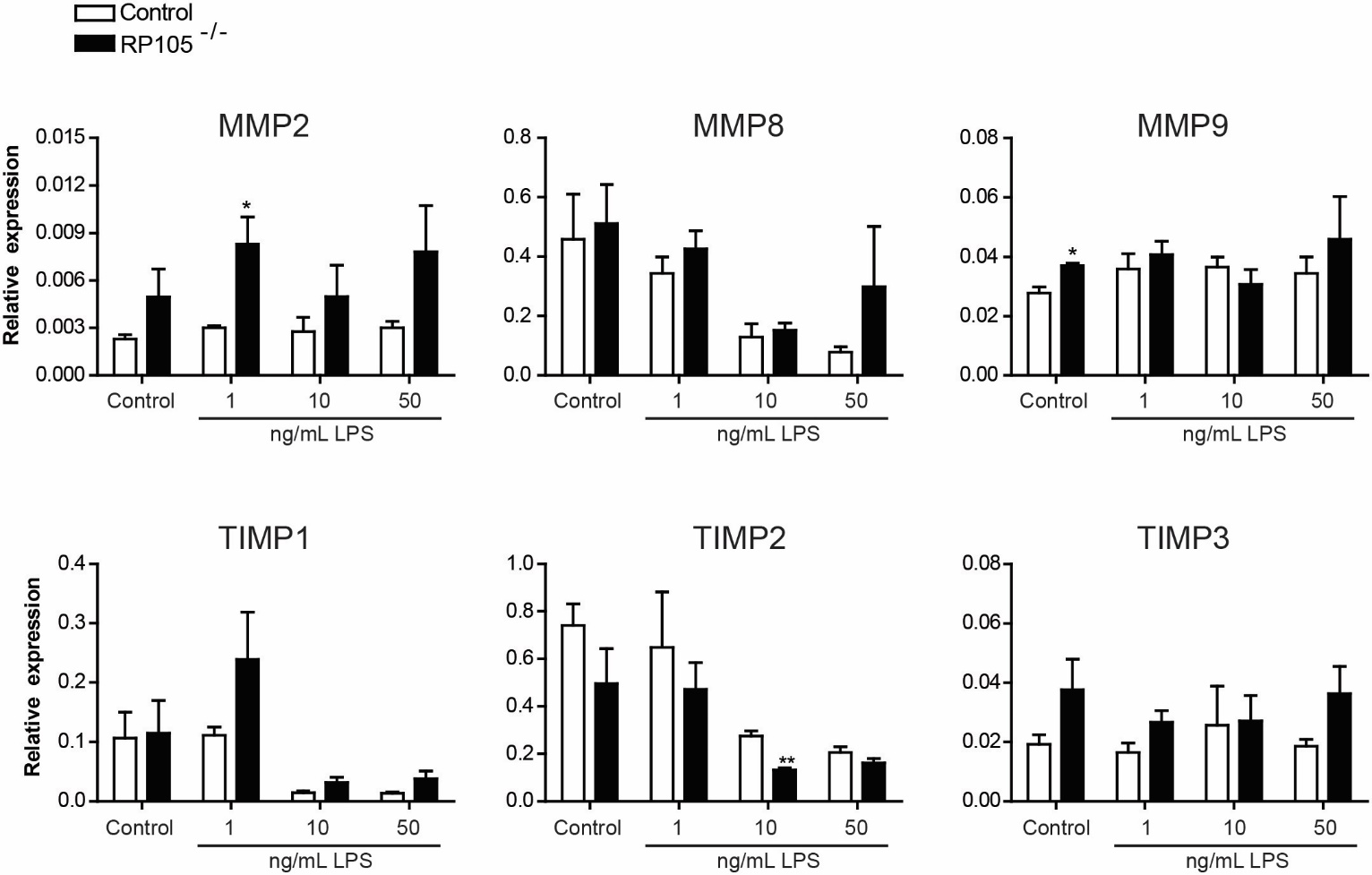


Supplemental Figure 8. Secretion of a) CCL2, b) IL-6 and c) TNFα did not differ between bone marrow derived macrophages derived from control or RP105 deficient mice.


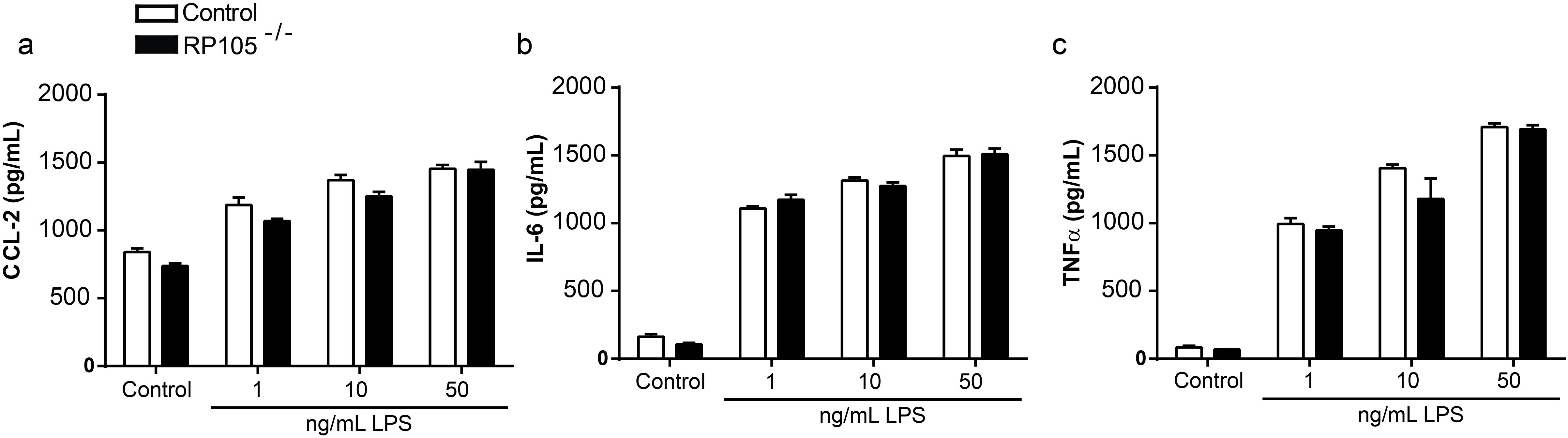


Supplemental Figure 9. Bone marrow derived mast cells express RP105 and TLR4 on mRNA (a) and protein level (b).


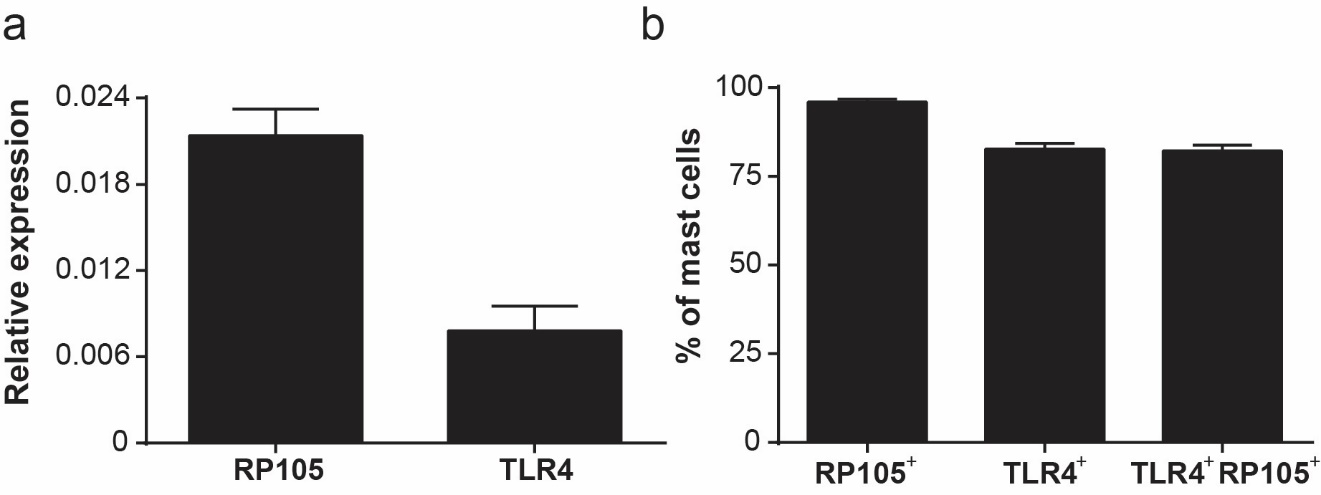


**Supplemental Figure 10.** Representative pictures of perivascular mast cells in a resting state (left panel) and in an activated state (right panel), with granules clearly surrounding the mast cell.


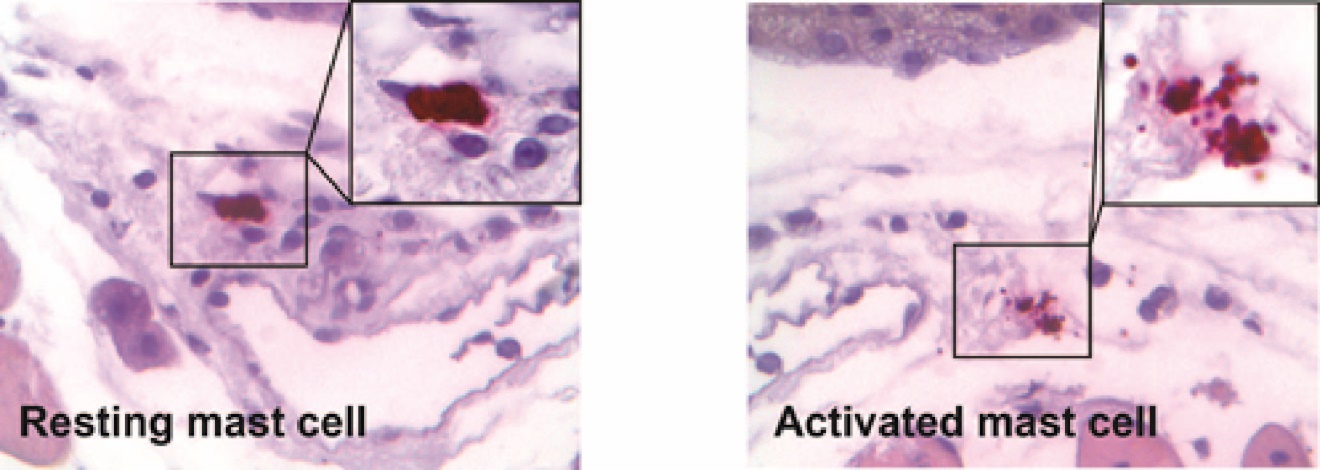

Supplement: Supplementary Information [file srep24248-s1.doc]
